# Correlation of radiographic and histopathological changes with IL-17 and advanced oxidation protein products in knee osteoarthritic individuals with metabolic syndrome

**DOI:** 10.1515/iss-2024-0037

**Published:** 2025-02-10

**Authors:** Uzma Naseer Awan, Rizwana Sanaullah Waraich, Syed Shahid Noor, Iftikhar Ahmed Siddiqui, Ruqaya Nangrejo

**Affiliations:** Department of Anatomy, Baqai Medical University, Karachi, Pakistan; Biomedical Research Center, Department of Biomedical & Biological Sciences, Sohail University, Karachi, Pakistan; Department of Orthopedics, Medicare Cardiac & General Hospital, Karachi, Pakistan; Baqai Medical University, Karachi, Pakistan; Department of Physiology, Baqai Medical University, Karachi, Pakistan

**Keywords:** metabolic syndrome, infrapatellar fat pad, advanced oxidation protein products, interleukin-17, inflammation, oxidative stress

## Abstract

**Objectives:**

Recent studies show that osteoarthritis and metabolic syndrome (MetS) represent significant global health concerns, sharing common pathological processes involving inflammation and oxidative stress. The study aimed to compare the radiological and histological severity of osteoarthritis in patients with and without MetS and further correlates them with oxidative stress and inflammatory markers in serum and synovial fluid (SF). Hypothesis: The study hypothesized that IL-17 and advanced oxidation protein products (AOPPs) are correlated with OA severity and progression in MetS patients.

**Methods:**

This cross-sectional study included 78 patients of advanced knee osteoarthritis, 40 with MetS and 38 without, matched for age. Clinical history and anthropometric measurements were recorded, and presurgical knee X-rays were evaluated using the Kellgren–Lawrence system. Histological grading of hematoxylin & eosin stained infrapatellar fat pad (IFP) and cartilage sections was performed. AOPPs and Interleukin-17 levels were measured in serum and SF, employing sandwich enzyme-linked immunosorbent assay.

**Results:**

In the MetS group, the severity of osteoarthritis was higher compared to non-MetS group, as evidenced by histological evaluation of the articular cartilage and IFP (p<0.05). The histological grading of IFP demonstrated positive correlation (p<0.05) with histological cartilage grade. Additionally, it exhibited a positive correlation with interleukin-17 and AOPPs in both SF and serum (p<0.05). While histological cartilage grade showed a positive correlation with AOPPs concentration in the serum and SF (p<0.05).

**Conclusions:**

MetS accelerates osteoarthritis progression, and positive correlation between molecular markers and histological severity suggests the contribution of inflammation and oxidative stress in the disease’s pathogenesis.

## Introduction

Osteoarthritis (OA) is the most widespread degenerative and debilitating joint disorder, affecting over 250 million individuals globally, with a predominant impact on the elderly population [1]. It is marked by the gradual loss of articular cartilage, reduction in space between joint bones, osteophyte formation, and synovial inflammation. OA usually occurs in the knee, hip, and hand joints [2].

Osteoarthritis exhibits a complex and multifaceted etiology, with various identified risk factors like gender, age, body weight, injuries to joint, metabolic conditions, inflammation, and genetic contribution [1], 3]. Recently, metabolic syndrome (MetS) linked to osteoarthritis has been suggested as a distinct OA phenotype [1].

As per the guidelines of the International Diabetes Federation, the occurrence of metabolic syndrome is 33.2 %, whereas the National Cholesterol Education Program guidelines indicate a prevalence of 23.9 % [4]. A causal connection between OA and MetS has been established, given their shared pathogenic pathway leading to increased inflammation and oxidative stress [3].

Several cross-sectional and cohort studies have identified metabolic disorders such as hypertension, dyslipidemia, and hyperglycemia as potential risk factors for osteoarthritis [5], [6], [7]. However, these findings lack consistency and warrant further investigation. While most research has proposed an association between individual metabolic disorders and OA [1], studies regarding the synchronous effects of MetS and its components on knee OA are limited and necessitate further exploration [8].

Mounting evidence highlights the link between osteoarthritis pathology and inflammatory markers, like interleukin (IL)-1β, tumor necrosis factor alpha (TNF-α), IL-6, and IL-17 [2], 9]. IL-17, predominantly produced by Th17 cells, accelerates cartilage degradation through the upregulation of matrix-degrading enzymes like matrix metalloproteinases (MMPs) and ADAMTS, as well as inducible nitric oxide synthase [10]. Currently, the infrapatellar fat pad (IFP), a collection of fatty tissue situated intra-articularly but extra synovially in the knee joint, is gaining attention due to its substantial role in knee osteoarthritis (OA) [11]. The IFP plays a role in OA by generating proinflammatory cytokines that can penetrate the articular cartilage through the synovial fluid, thereby stimulating the expression of catabolic mediators [12]. Recent research findings indicate that progression of OA is closely associated with oxidative stress and reactive oxygen species (ROS) [13]. AOPPs are recognized as novel indicators of oxidative stress, and it has been observed that they accelerate cartilage degeneration in rabbit OA models by upregulating the protein expression of matrix metalloproteinases-3 (MMP-3) and MMP-13 in the synovium [14]. Another study showed that AOPPs contribute to OA progression by increasing TNF-α and IL-1β expression in chondrocytes via the NADPH oxidase 4-p38-MAPK pathway, leading to cartilage degeneration in rodent models [15]. These mechanisms link oxidative stress and inflammation to OA in MetS patients.

To our knowledge, this is the first study that has comprehensively assessed the link between metabolic syndrome and OA by correlating radiological and histopathological grading of cartilage and IFP with levels of AOPPs and IL-17 in OA patients of MetS and non-MetS group. This understanding is crucial for identifying biomarkers and devising early prevention or treatment strategies to halt or delay OA.

## Materials and methods

### Patient selection

Total 78 patients with advanced knee osteoarthritis, meeting the standards established by the American College of Rheumatology (ACR) [16] and undergoing total knee replacement surgery, were enrolled in the study at the Orthopedic Surgery Department of Medicare Cardiac and General Hospital, Karachi, from December 2022 to October 2023. The sample size was determined using the software “Select Statistical Services,” with the study power set at 80 % and a confidence interval of 95 %. The patients were divided into two groups: 40 in the MetS group and 38 in the non-MetS group. The study was granted approval by Institutional Review & Ethics Board (IREB) and Board of Advance Studies & Research (BASR) of Baqai Medical University, Karachi. Written agreement was acquired from the participants in compliance with the Declaration of Helsinki. The inclusion criteria comprised diagnosed cases of advanced knee OA patients with metabolic syndrome undergoing knee replacement surgery. The exclusion criteria ruled out patients who had a history of previous joint surgery or trauma, and those with preexisting conditions like various forms of arthritis, cancer or chronic inflammatory diseases, and patients currently taking steroids or anticancer medicines.

The diagnosis of metabolic syndrome followed the criteria outlined by the National Cholesterol Education Program Adult Treatment Panel III (ATPIII) for Asians, which defines metabolic syndrome based on the presence of three or more specified risk factors (components): higher waist circumference (102 cm (≥40 in) for men, 88 cm (≥35 in) for women), increased triglycerides (TG) (≥150 mg/dl), low high-density lipoproteins (HDL) cholesterol (<40 mg/dl in men, < 50 mg/dl in women), high BP (≥130/85 mmHg), hyperglycemia (≥100 mg/dl) [17].

### Demographic and clinical parameters

A detailed demographic and clinical history of the patient along with various anthropometric measurements were carefully recorded (Table 1) maintaining the patient confidentiality. Patients were classified into three groups as per WHO classification of obesity according to their body mass index (BMI): normal weight (BMI 18.5–24.9 kg/m^2^), overweight (BMI 25–29.9 kg/m^2^), and obese (BMI≥30 kg/m^2^) [18].

**Table 1:** Comparison of demographic and clinical parameters between studied groups.

Parameters	Non-MetS (n=38)	MetS (n=40)	p-Value
	n (%)	n (%)	
Gender			0.06
Male	10 (26.3 %)	4 (10 %)	
Female	28 (73.7 %)	36 (90.0 %)	
BMI levels			0.035^a^
Normal weight	8 (21.1 %)	2 (5.0 %)	
Overweight	14 (36.8 %)	11 (27.5 %)	
Obese	16 (42.1 %)	27 (67.5 %)	
Joint disease duration≥10 years	16 (42.1 %)	22 (55.0 %)	0.25
Abnormal waist	30 (78.9 %)	40 (100.0 %)	0.002^a^
Diabetes	2 (5.3 %)	28 (70.0 %)	<0.001^a^
Hypertension	16 (42.1 %)	40 (100.0 %)	<0.001^a^
Heart disease	0 (0.0 %)	4 (10.0 %)	0.04^a^
Elevated triglycerides	0 (0.0 %)	16 (40.0 %)	<0.001^a^
Low HDL	10 (26.3 %)	35 (87.5 %)	<0.001^a^

	**Mean** **± SD**	**Mean** **±** **SD**	

Age, years	63.9 ± 7.7	63.9 ± 6.8	0.99
Waist circumference (inch)	41.3 ± 5.2	42.3 ± 4.3	0.39
BMI I (kg/m^2^)	29.6 ± 6.7	32.2 ± 4.3	0.04^b^
Triglyceride (mg/dl)	78.0 ± 23.6	127.3 ± 55.1	<0.001^b^
HDL cholesterol (mg/dl)	52.3 ± 11.0	39.1 ± 14.0	<0.001^b^

^a^p<0.05 was considered statistically significant using Pearson Chi Square test, ^b^p<0.05 was considered statistically significant using Independent sample t-test.

### Sample collection

#### Serum and SF

From each patient, a fasting venous blood sample of 5 mL was collected in serum separator tubes and then centrifuged at 3,000 rpm. SF was aspirated from knee joint during surgery and centrifuged at 4,000 rpm for 10 min to eliminate cellular debris. The resulting samples were kept at −80 °C until further analysis [19].

Blood samples were employed for measuring fasting blood glucose levels and serum concentrations of triglycerides (TG) and high-density lipoproteins (HDL).

#### Radiographic assessment

For each patient, preoperative radiological images of the knee joint in anteroposterior (AP), lateral projection, and sky view were obtained and were assessed in a blinded fashion by a radiologist, according to Kellgren and Lawrence (KL) score [20].

### Tissue processing and histological analyses

#### IFP and articular cartilage

Tissue samples including articular cartilage and IFP were obtained during knee replacement surgery. For histological examination, tissues were fixed in 10 % neutral buffered formalin for 72 h. Cartilage samples underwent additional decalcification with 10 % formic acid for 72–96 h before further processing.

During tissue processing, tissue specimens underwent dehydration in graded ethanol, followed by clearing in xylene, and were ultimately embedded in paraffin while preserving their anatomical orientations [21]. Tissue sections 5–8 μm thick were prepared using KD-2260 Rotary microtome (KEDEE, China) and stained with hematoxylin and eosin (H&E). The images were captured under a light microscope (Nikon Ti2 inverted microscope, Japan) after calibration.

### Histopathological evaluation

Articular cartilage was histologically graded according to the Osteoarthritis Research Society International (OARSI) score system [22].

The scoring of microscopic features of the IFP was graded as under:

– Grade 0=no presence of lymphocytic infiltration;
– Grade 1=presence of perivascular mononuclear cell infiltration;
– Grade 2=both perivascular and interstitial mononuclear cell infiltration.

For analyzing vascularization in the IFP, a number of vessels were counted in four randomly selected fields from a single tissue slice at a magnification of × 200. Subsequently, the mean number of vessels was calculated [23].

### Morphometric analysis

Images of H&E-stained sections of IFP captured at × 100 magnification were converted into binary images (black and white) for analysis using a microscope (Optika, Italy) equipped with specialized imaging and measuring software, Optika ProView software. Adipocytes were represented by white regions, while the black regions represented the boundaries, facilitating the calculation of adipocyte number and area [24].

### Immunoassays measurements

The enzyme-linked immunosorbent assay kit from Human AOPPs Abbexa, Cambridge, and the Human IL-17 ELISA kit (R&D System, Minneapolis, MN, USA) were employed to measure the concentrations of AOPPs and IL-17 in serum and synovial fluid, following the instructions provided by the manufacturer.

### Statistical analysis

SPSS statistical software, IBM-SPSS version 23.0 was used to perform the data analyses. Independent sample t-test was utilized for normally distributed values, while the Pearson chi-squared test was employed to compare observed frequencies of categorical data. Spearman correlation coefficients were used for ordinal data, and Pearson correlation coefficients were used for continuous numerical data to evaluate correlations after adjusting for age and gender, with statistical significance at a p-value <0.05.

## Results

### Demographic and clinical characteristics of patients

In our study, we observed a higher frequency of OA in females, with mean ages of 63.9 ± 7.7 in the non-MetS group and 63.9 ± 6.8 in the MetS group (p>0.05) (Table 1). This finding is supported by various studies suggesting that beyond the age of 50, the incidence of OA in women increases more rapidly than in men, indicating a potential influence of menopause on OA progression [25]. Furthermore, OA patients in the MetS group exhibited higher mean BMI and triglyceride levels (p<0.04, p<0.001, respectively), whereas mean HDL levels were significantly less in the OA with MetS patients compared to the non-MetS group patients (p<0.001), as determined by the Independent sample t-test (Table 1).

### Radiological and histopathological grading

**Figure 1:**
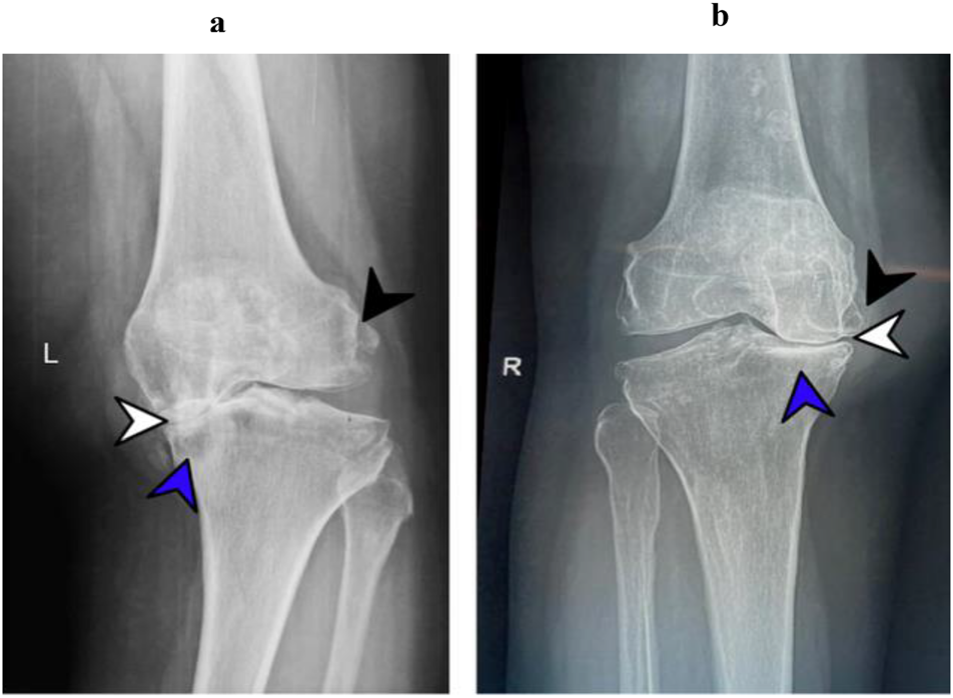
Anterior-posterior (AP) radiographs of the knee from OA patients. (a) MetS group has marked osteoarthritic changes exhibiting, loss of medial joint space (white arrow head), moderate osteophyte formation (black arrow head), and discrete sclerosis with cysts formation (blue arrow head). (b) Non-MetS group shows advanced narrowing of joint space (white arrow head), osteophyte formation (black arrow head), and discrete subchondral sclerosis (blue arrow head).

In this study, the OARSI scoring system for cartilage degradation and the Kellgren–Lawrence (KL) grading system for radiological changes were used, as various studies have demonstrated their complementary roles in assessing different aspects of joint degeneration [15], 26]. The Pearson Chi-Square test revealed positive association of histological cartilage grade with IFP grade (p<0.05) between the studied groups. Conversely, KL grading demonstrated consistent results across groups, indicating an insignificant association between them (p<0.37) (Table 2). These findings align with those of Yasuda et al. who observed no significant association between radiographic changes in knee OA and individual or cumulative components of MetS [27]. However, contrary to these results, another study has reported significantly advanced radiological changes in the MetS group [28] (Figure 1).

### Association of number of MetS components with histological and radiological severity of OA

The radiological (p=0.667) and histological grading of cartilage (p=0.061) in OA patients did not show a significant association with the number of components present in metabolic syndrome (MetS), likely due to the small sample size. In contrast, histological grading of the IFP significantly increased in OA patients with an increasing number of MetS components (p=0.004) using Pearson’s Chi-Square test. These results are in line with a study that demonstrates significant association of metabolic disorders with OA severity [1].

### Histological features of IFP and articular cartilage

Histological analysis of IFP in the MetS group revealed higher grades of inflammation, extensive fibrosis, and morphometric changes, such as increased vascularization (p=0.00) (Figure 3c) and adipocyte size (p=0.00) (Figure 3e), along with a diminished adipocyte cell count (p=0.00) (Figure 3d) compared to the OA non-MetS patients, as determined by the independent sample t-test (Table 2). These findings are supported by an earlier study reporting similar changes in the IFP of OA patients compared to patients undergoing anterior cruciate ligament (ACL) reconstruction [24].

**Figure 2:**
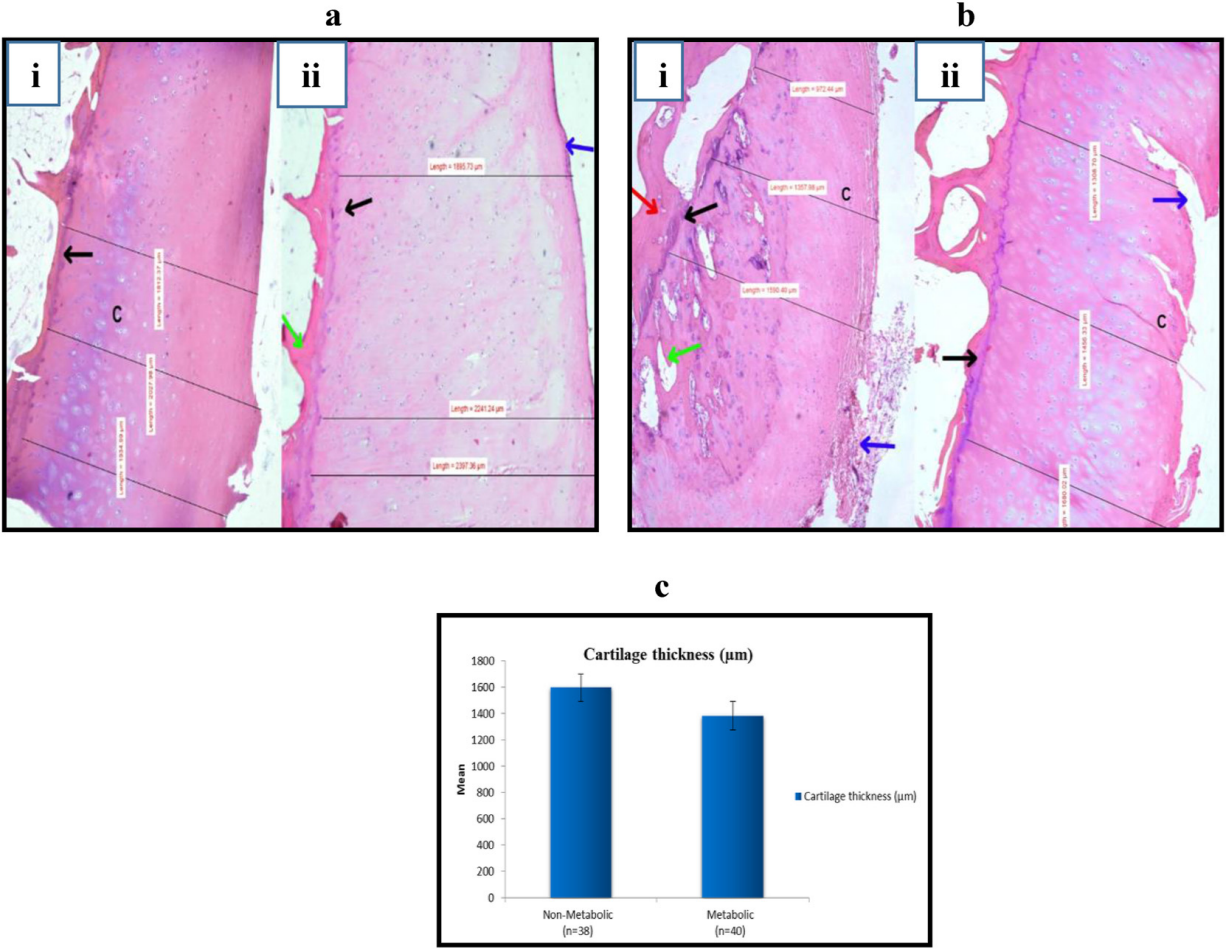
Histological examination of articular cartilage in OA patients. (2a) Non-MetS group indicates (i,ii) increased cartilage thickness (indicated on the scale), decreased surface erosion (blue arrow), intact tidemark (black arrow), organized chondrocytes (c), and a thinner subchondral plate (green arrow); (2 b) MetS group demonstrates (i) decreased cartilage thickness, more surface erosion (blue arrow), preserved tidemark (black arrow), and subchondral plate thinning (green arrow). (Ii) shows surface fissuring (blue arrow), tide mark duplication in early stages and presence of cell clusters near surface (c) (H & E stain, magnification×40). (2c) Comparison of articular cartilage thickness in OA patients between the MetS and non-MetS groups, (p=0.07).

**Figure 3:**
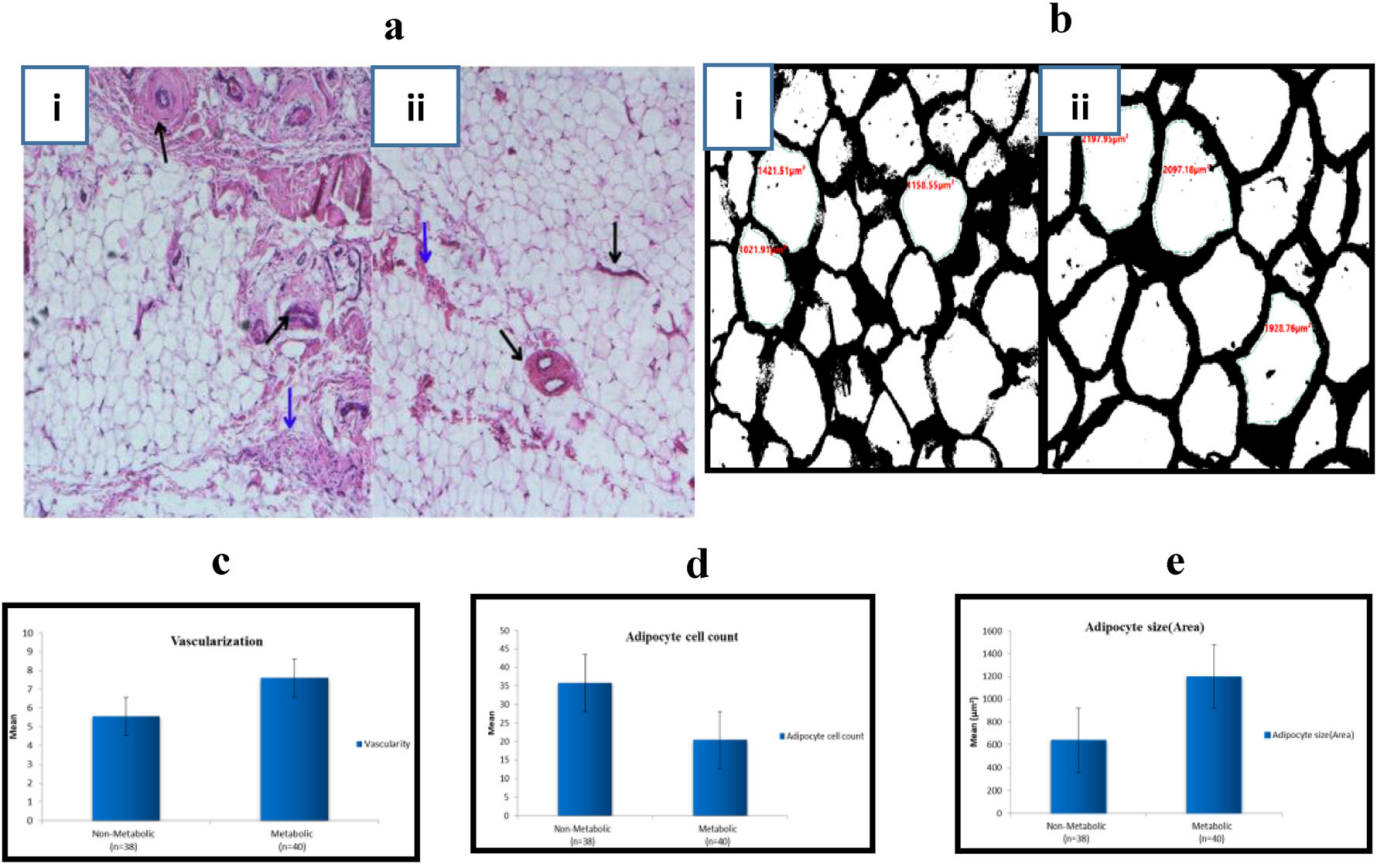
Histological analysis of infrapatellar fat pad (IFP) between studied groups in OA patients. (a) Histological characterization of IFP; (i) MetS group has increased collagen fibers density (blue arrow) and vascularization (black arrow). In contrast, (ii) non-MetS group shows lower collagen fibers density (blue arrow) distributed among clusters of adipocytes and fewer blood vessels (black arrow) (H & E stain, magnification×40). (b) Adipocyte morphology evaluation in (i) non-MetS group and (ii) MetS group shows an enlargement in adipocyte size and a reduction in the number of adipocytes in MetS group (p<0.05), (3c) comparison of number of vessels in the IFP of OA patients between studied groups, (p<0.05). (3d and 3e) Comparison of adipocyte cell count and adipocyte size in the IFP of OA patients between studied groups (p=0.00).

**Table 2:** Comparison of radiological and histopathological parameters between non-MetS and MetS groups.

Parameter	Non-MetS (n=38)	MetS (n=40)	p-Value
	n (%)	n (%)	
**Radiological KL grade**			0.37
Grade-2	14 (36.8 %)	15 (37.5 %)	
Grade-3	24 (63.2 %)	25 (62.5 %)	
**Histological cartilage grade**			0.005^a^
Grade-2	0 (0.0 %)	4 (10.0 %)	
Grade-3	12 (31.6 %)	4 (10.0 %)	
Grade-4	20 (52.6 %)	16 (40.0 %)	
Grade-5	6 (15.8 %)	16 (40.0 %)	
**Histological IFP grade**			0.002^a^
Grade-1	24 (63.2 %)	11 (27.5 %)	
Grade-2	14 (36.8 %)	29 (72.5 %)	

	**Mean** **± SD**	**Mean** **± SD**	

Vascularization	5.55 ± 0.9	7.6 ± 0.8	<0.001^a^
Cartilage thickness, µm	1,595.2 ± 604.7	1,381.6 ± 410.1	0.07
Adipocyte cell count	35.8 ± 5.3	20.4 ± 4.4	<0.001^a^
Adipocyte size (area)	636.1 ± 111.3	1,197.8 ± 343.5	<0.001^a^

^a^p<0.05 was considered statistically significant. Pearson’s Chi-Square test was used for categorical data, and Independent sample t-test was used for mean comparisons.

In MetS group, elevated grades of cartilage degeneration (p<0.05) were noted (Figure 2b, Table 2). However, mean difference in cartilage thickness was not significant among the groups (p=0.070) (Table 2, Figure 2). Pragasam and Venkatesan observed comparable changes in the articular cartilage in an animal-based study, associating them to MetS [29]. We also observed thickening of the subchondral plate, along with the infiltration of vascular elements from the subchondral bone and adjacent marrow space into the calcified cartilage. This process led to the duplication of the tidemark and localized thinning of the cartilage (Figure 2b). These findings are in accordance to earlier studies that have reported similar changes in the cartilage structure [14], 30].

### Spearman’s rank correlation between histological data of the IFP and articular cartilage with molecular markers

BMI (p=0.000) and histological cartilage grade (p=0.007) showed positive correlations with the histological grade of the IFP of overall cohort. Moreover, IFP histological grading exhibited positive correlations with vascularization (p=0.001), and adipocyte size (p=0.013) while demonstrating a negative correlation with adipocyte cell count (p=0.007) and cartilage thickness (p=0.003) of overall cohort (Table 3).

**Table 3:** Spearman’ rank correlation analysis of BMI, radiological, and histological grades among studied parameters.

Parameters	BMI	KL grade	Cartilage grade	IFP grade
BMI	1	0.150	0.206	0.538^b^
Radiological KL grade	0.150	1	0.152	0.009
Histological cartilage grade	0.206	0.152	1	0.304^b^
Histological IFP grade	0.538^b^	0.009	0.304^b^	1
Vascularization	0.272^a^	0.001	0.284^a^	0.367^b^
Cartilage thickness	−0.160	0.078	0.052	−0.328^b^
Adipocyte cell count	−0.383^b^	−0.011	−0.237^a^	−0.303^b^
Serum (AOPPs)	0.272^a^	−0.047	0.288^b^	0.316^b^
SF (AOPPs)	0.352^b^	−0.004	0.239^a^	0.359^b^
Serum (IL-17)	0.249^a^	0.060	0.180	0.241^a^
SF(IL-17)	0.246^a^	0.016	0.067	0.315^b^

^a^Correlation considered statistically significant with p<0.05, ^b^Correlation was considered highly statistically significant with p<0.01.

Histological cartilage grade demonstrated a positive correlation with AOPPs levels in both serum and SF (p=0.011 & p=0.035 respectively), while IFP histological grading showed positive correlation with AOPPs levels in both SF (p=0.001) and serum (p=0.005) and IL-17 levels in both SF (p=0.005) and serum (p=0.033) in overall cohort (Table 3). There was no significant correlation of radiological grades with histological grades of cartilage (p=0.184) and IFP (p=0.935).

### Correlation between molecular markers in serum and SF using Pearson correlation

**Figure 4:**
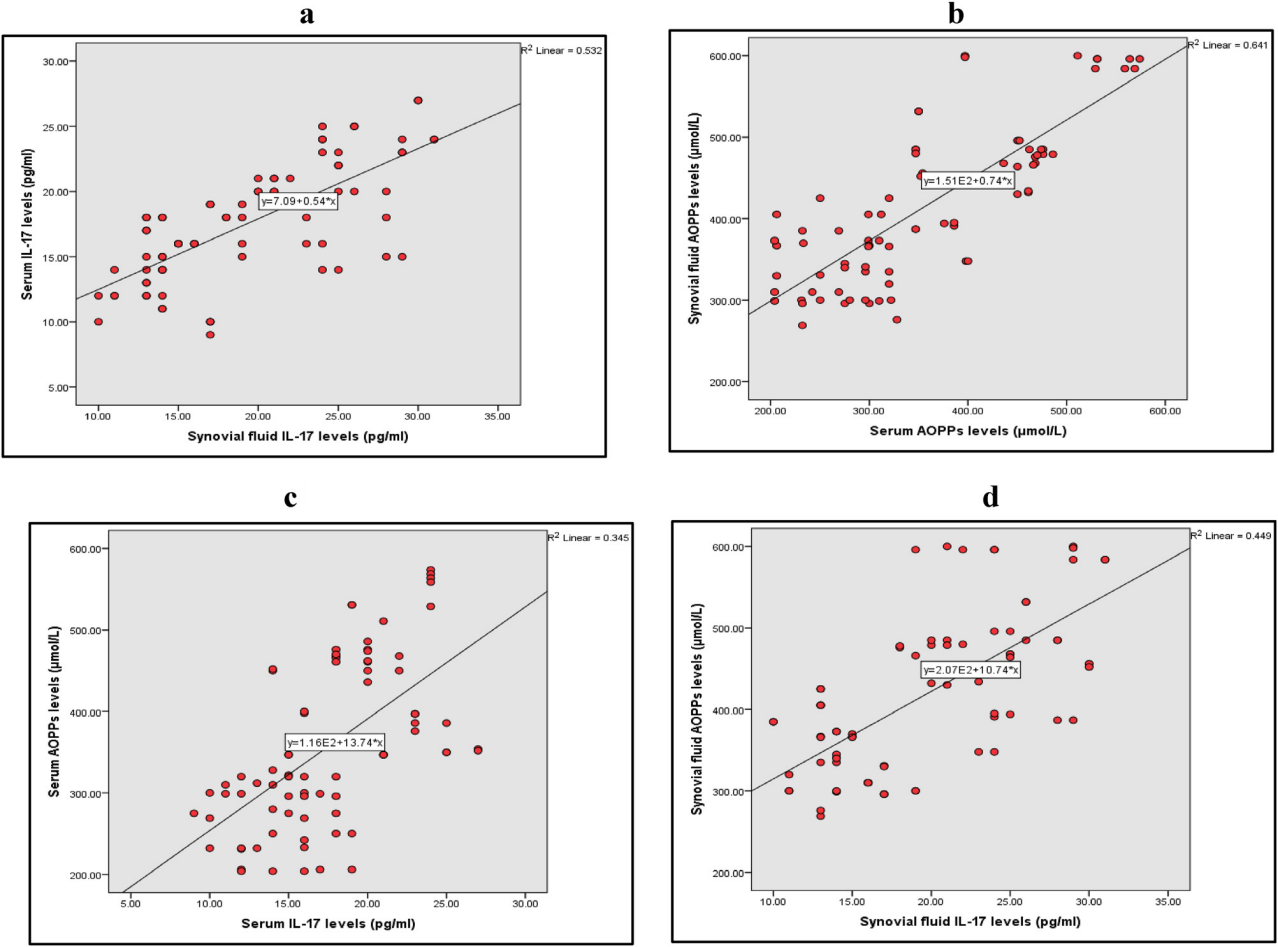
Graphical representation of correlation analysis of various markers among the investigated groups. (a) IL-17 levels in SF and serum (p<0.05). (b) AOPPs levels in serum and SF (p<0.05). (c) Levels of IL-17 and AOPPs in the serum (p<0.05. (4d) Levels of IL-17 and AOPPs in SF (p<0.05) using Pearson correlation analysis.

A significantly positive correlation was found between serum IL-17 and SF IL-17 (p=0.00) (Figure 4a) and, similarly, a significantly positive correlation was observed between serum AOPPs and SF AOPPs (p=0.00) (Figure 4b).

Furthermore, serum IL-17 exhibited a positive correlation with AOPPS in both serum (p=0.00) (Figure 4c) and SF (r=0.682. p=0.00). Likewise, SF IL-17 showed a positive correlation with the AOPPs in both serum (r=0.693. p=0.00) and SF (p=0.00) (Figure 4d).

### Logistic regression analysis of samples from OA patients with MetS

In univariate model, cases of MetS group are more likely associated with obesity, high TG levels, low HDL, diabetes, high IFP grade, increase in vascularization, decrease in adipocyte cell count, increase in adipocyte size (area), high SF AOPPs levels, high SF IL-17 levels, and high serum IL-17 levels (Table 4).

**Table 4:** Risk estimation of metabolic samples using binary logistic regression.

Parameters	Univariate odds ratio (95 % C·I.)	Multivariate^¥^ odds ratio (95 % C·I.)
BMI overweight	3.14 (0.55–17.8)	2.96 (0.50–17.2)
BMI obese	6.74^a^ (1.27–35.7)	5.40 (0.97–30.1)
TG	1.02^a^ (1.01–1.04)	1.02^a^ (1.01–1.04)
Low HDL	19.5^a^ (6.00–63.9)	30.8^a^ (7.15–133.3)
Diabetes	41.9^a^ (8.68–203.1)	56.7^a^ (9.93–324.5)
IFP grade	4.51^a^ (1.73–11.7)	4.82^a^ (1.68–13.8)
Vascularization	28.5^a^ (5.01–162.1)	128.0^a^ (6.81–2,403.2)
Adipocyte cell count	0.23^a^ (0.07–0.66)	0.20^a^ (0.04–0.87)
Adipocyte size (area)	1.01^a^ (1.00–1.02)	1.01^a^ (1.00–1.02)
SF(AOPPs)	1.04^a^ (1.02–1.06)	1.05^a^ (1.02–1.09)
SF(IL-17)	5.77^a^ (1.58–21.0)	3,378 (0.00–7,729)
Serum (IL-17)	1.93^a^ (1.45–2.57)	1.95^a^ (1.44–2.64)

Dependent variable: metabolic group. ^a^Odds ratio considered statistically significant with p<0.05. ¥: Model was adjusted for age and gender.

Whereas in multivariate model after adjusted with age and gender, it was found that MetS group samples were more likely associated with high TG, low HDL, diabetes, high IFP grade, increased IFP vascularization, adipocyte cell count were less likely associated with, increased adipocyte size (area), high SF levels of AOPPs, and high serum levels of IL-17. Above findings showed statistically significant p-value (p<0.05) (Table 4).

## Discussion

Several epidemiological and cross-sectional studies have shown a bidirectional link between MetS and osteoarthritis (OA), leading to earlier onset and accelerated disease progression [31], 32]. In the current study, patients of MetS group displayed increased BMI (p<0.04) and a higher prevalence of comorbidities compared to those without MetS (p<0.001). Moreover, binary logistic regression analysis indicated significant associations of MetS with obesity, DM, high TG, and low HDL (p<0.05). These results align with various epidemiological and clinical studies representing similar alterations in demographic parameters and also highlighted association between knee OA and MetS with higher levels of pain, functional impairment, and radiological damage [26], 28].

Exploring the histological characterization of IFP in OA patients, including inflammation, adipocyte morphology, and extracellular matrix alterations, is crucial for understanding its role in OA development, much like the analysis conducted for other tissues such as the synovial membrane and cartilage [23], 26]. In MetS group, a positive correlation was observed between BMI and histological IFP grades, consistent with findings from a recent study conducted by Belluzzi et al. [24].

In the current investigation, the observed positive correlation between histological cartilage grade and serum and SF, AOPPs levels indicates a potential role of oxidative stress in both accelerated cartilage degradation and increased apoptosis. This finding is reinforced by another study, wherein AOPPs were implicated in inducing cell death in human chondrocytes *in vitro*, mediated by ROS-dependent PARP-1 activation [33]. To date, there exists a research gap concerning the correlation between molecular markers and the histological grade of IFP. Our study reveals a positive association between IFP histological grading and the levels of AOPPs and IL-17 in serum and SF, suggesting their potential involvement in triggering significant inflammatory and morphometric changes in IFP. This observation resonates with an earlier study where AOPPs were implicated in inducing inflammatory and profibrotic processes in kidneys [34]. An animal-based study further supports these findings by highlighting the profibrogenic role of IL-17 on both inflammatory and liver resident cells [35]. Clinical studies have implicated IL-17 signaling in the fibrogenesis of organs like the lungs and skin as well as in driving inflammation through proinflammatory cytokines that cause tissue damage. Elevated AOPPs levels have also been linked to the progression of OA and other degenerative diseases, chronic kidney and cardiovascular diseases, where oxidative stress is a key factor. These findings highlight the close interplay between oxidative stress and inflammation in creating a destructive environment in osteoarthritic joints [15]. Current study also highlighted a positive correlation between the inflammatory marker IL-17 and the oxidative stress marker AOPPs in biofluids, indicating the interdependence of these two pathological processes in OA progression. Previous research has also shown a comparable association, indicating that increased levels of inflammatory markers in OA joints lead to oxidative stress and cause joint damage through the expression of matrix-degrading proteases [3], 19].

## Conclusions

Oxidative stress and inflammation, fundamental processes underlying metabolic syndrome, play a crucial role in the pathogenesis of OA. Biomarkers such as AOPPs and IL-17 may aid in assessing disease severity and therapeutic efficacy. We recommend further research, including interventional studies targeting biomarkers like IL-17 and AOPPs, to explore their therapeutic potential. Controlled trials are needed to refine their diagnostic and prognostic value, and future longitudinal studies should confirm and strengthen these findings.

A limitation of this study is its cross-sectional design, which prevents the establishment of causality. Future longitudinal or experimental studies are needed to better understand causal pathways. Additionally, comprehensive studies across biofluids are essential to explore the interrelationships among biomarkers and their potential for improving patient care and precision medicine.
